# Empower Our Growth as Athletes: Voices of Swedish Athletes With Intellectual Disability

**DOI:** 10.1111/jar.70133

**Published:** 2025-09-30

**Authors:** Johanna Oskarsson, Eva Flygare Wallén, Kim Wickman, Marie Lund Ohlsson

**Affiliations:** ^1^ The Swedish Parasport Federation Stockholm Sweden; ^2^ Department of Health Sciences, Swedish Winter Sport Research Centre Mid Sweden University Östersund Sweden; ^3^ Karolinska Institutet (KI), Department of Neurobiology, Care Sciences and Society (NVS) Division of Occupational Therapy Research Group Health in Everyday Life Among People With Neurological Disorders (HELD) Stockholm Sweden; ^4^ Health and Social Care Administration, Municipality of Östersund Östersund Sweden; ^5^ Department of Education Umeå University Umeå Sweden; ^6^ Department of Physiology, Nutrition and Biomechanics The Swedish School of Sport and Health Sciences (GIH) Stockholm Sweden

**Keywords:** inclusion, motivation, para sports, physical activity, sense of belonging

## Abstract

**Background:**

People with intellectual disability participate in organised sports to a lesser extent than people in general. The aim of this study was to explore the motivation for engaging in sports among athletes using the theoretical framework of sense of belonging.

**Method:**

Semi‐structured online interviews were carried out with athletes (*N* = 15, 8/7 women/men, 29.5 ± 9 years) with intellectual disabilities.

**Results:**

The main theme ‘Empower me to grow as an athlete—adapt the support to help me develop my sport skills and social aspects’ showed that motivation, competence, perception, and opportunities were important factors for engaging in sports. Support was an important factor for creating adapted opportunities.

**Conclusion:**

Athletes with intellectual disabilities have motivation, want to feel competent, and to be athletes who belong in sports and develop as such. They also appreciate coaches giving adapted support that enhances understanding and promotes inclusion in the sport community.

**Summary**

- This study offers unique insights on sports inclusion for athletes with intellectual disabilities, focusing on the facilitators of inclusion. The study shows the feasibility of including voices of people with intellectual disabilities in research, instead of only exploring the views of parents, support staff or coaches.
- People with intellectual disability want to feel like athletes in their sport and be treated as such.
- Athletes with intellectual disability appreciate support from coaches that promotes a sense of belonging in the sport context, regarding both sport skill development and social aspects.
- There are limitations in the sport development pathway for people with intellectual disabilities. Athletes experience a sense of exclusion related to fewer competition opportunities and limitations in how far one can progress in certain sports.

## 1 Background

The meaning of sport participation for people with intellectual disabilities needs more attention (Hassett et al. 2024; Brighton et al. 2022). In general, sport participation for people with intellectual disabilities brings many health benefits (Hassett et al. 2024), in terms of physical health and fitness (Gallotta et al. 2024), social aspects, and autonomy (Farrell et al. 2004). The individual motives for people with intellectual disabilities to participate in sports are multiple, such as fun/enjoyment, social aspects, performance, achieving success, and well‐being (Harada and Siperstein 2009), which are similar to motives for sport participation among the general population of athletes (Domínguez‐Amorós and Aparicio‐Chueca 2020).

In Sweden, around 1%–1.5% of the population have an intellectual disability (Folkhälsomyndigheten 2024). Intellectual disability is characterised by a significant limitation in intellectual functioning and adaptive abilities within the areas of conceptual, social, and practical skills (Schalock et al. 2021). Studies show that, on average, people in this group engage in less physical activity (Dairo et al. 2016) and experience a higher burden of health conditions, especially when the disability is more severe, than the population as a whole (Flygare Wallén et al. 2018; Kinnear et al. 2018). Also among athletes with intellectual disabilities, health problems are common. A study comparing Special Olympics (SO) athletes and non‐athletes with intellectual disability in terms of physical health showed that athletes had higher levels of physical activity, but both groups still displayed risk factors for physical health problems (Oskarsson et al. 2023; Fjellström et al. 2024).

Participation in organised sport activities is lower for people with intellectual disability compared to the general population (Borland et al. 2020; Robertson et al. 2018). The multifaceted barriers to participation in physical activity perceived by people with intellectual disability are personal (health problems, preference for an inactive lifestyle), social (lack of inclusion and adapted physical activity options), and environmental (Jacinto et al. 2021). However, people with intellectual disability are most often dependent on personal support from family or support staff to be physically active, and the role of personal support to promote physical activity is of high importance (Kreinbucher‐Bekerle et al. 2022; McGarty and Melville 2018). Personal support people often also perceive barriers for people with intellectual disability such as the disability itself, social aspects (lack of adapted physical activity programmes, lack of support, presence of stigma and social exclusion, lack of inclusion, transportation), acceptance of an inactive lifestyle, and limited financial resources (Jacinto et al. 2021). For participation in sports, the barriers are similar, that is, lack of support and fear of social exclusion, the feeling of not ‘fitting in’ (Hansen et al. 2023). Sport is often structured around normative expectations of physical and cognitive functioning, which can lead to feelings of exclusion when athletes do not match these standards (Svanelöv et al. 2020).

While there are a number of studies on the barriers to sport participation for people with intellectual disability, few have explored the facilitators for participation and what kinds of adaptations and support provided in sports clubs make people with intellectual disability feel a sense of inclusion and belonging in sports. A literature review by Geidne and Jerlinder (2016) on how sports clubs include young people with disabilities showed that there is a need for more research about what actually works regarding recruitment, coaching, and adapted training and support. When people with disabilities stay involved in sport, it is because there are effective strategies in place. A study of SO athletes in Canada revealed that the most important factors were the frequency of sports participation and the social aspects (friendship, psychosocial gains, and environmental support) (Weiss et al. 2020).

Research into the perspectives of people with intellectual disability is often conducted by interviewing family members and others in their network, such as their parents, teachers, coaches, or support staff. In a recent study done in Europe (Sakalidis et al. 2023), coaches were asked about the inclusion of athletes with intellectual disability in their sport club. The study highlighted the need for greater emphasis on the needs, motivations, and social context of all athletes in the club, that is, the importance of educating all athletes and club representatives to challenge the dominant norms and expectations within their sports culture, in order to create a social environment that actively supports a sense of belonging for all participants. Some studies interview a combination of athletes with intellectual disability and their parents or support persons (Grandisson et al. 2012), thereby making it hard to understand the perspectives of the athletes themselves.

### 1.1 Theoretical Approach

In this study, as part of the broader concept of inclusion, we explore when and how athletes with intellectual disability experience a sense of belonging within the context of sports clubs. Our analysis is guided by the integrative framework developed by Allen et al. (2021) for understanding and assessing belonging. According to Allen et al. (2021), belonging is defined as a subjective feeling of deep connection to surrounding systems, such as family, friends, social networks, or physical environments. The current study specifically focuses on the social aspects of sports participation perceived by people with intellectual disability. The study participants are active in sport clubs (organised sports) and either training together with people without disability (referred to as included activity), training in a disability‐specific group in a club where people without disability are also active (integrated activity), or active in a disability‐specific sports club (segregated activity).

Belonging is a basic human need linked to positive outcomes such as better social relationships and improved well‐being (Allen et al. 2021). Though subjective, the sense of belonging is shaped by social context and influenced by external factors. As individuals with intellectual disability often face personal difficulties regarding social and conceptual aspects, they may be especially sensitive. The Physical Activity for People with a Disability (PAD) model (van der Ploeg et al. 2004) shows how personal and environmental factors interact to create facilitators and barriers to activity. It emphasises the interplay between individual factors such as motivation, health, and self‐efficacy; environmental factors including access to facilities, equipment, and social support; and societal influences such as norms, policies, and institutional structures. By highlighting both barriers and facilitators, the model serves as a tool to promote inclusion, equity, and health through enhanced participation in physical activity. The current study investigates how and when athletes with intellectual disabilities experience belonging in sports clubs, keeping in mind disability‐specific aspects using the PAD model, by examining four interconnected components identified by Allen et al. (2021): competence, opportunities, motivations, and perceptions. First, in terms of competencies, the analysis focuses on the extent to which athletes with intellectual disabilities possess the skills and abilities necessary to foster connections and experience belonging. Second, opportunities for belonging are examined by identifying when and how the sports club environment facilitates social inclusion. Third, motivations are explored, with attention to the reasons athletes with intellectual disability seek to connect with others within or through the sports club. Lastly, perceptions are assessed to investigate how athletes interpret and evaluate their sense of belonging.

Therefore, the aim of this study was to explore motivation for engaging in organised sports using the theoretical framework of sense of belonging for athletes with intellectual disability. A secondary aim was to explore the athletes' need for support.

## 2 Method

### 2.1 Study Design

This qualitative study used semi‐structured individual interviews with athletes active in organised sports in Swedish sports clubs. The inclusion criteria were: 15–55 years old, training at least once a week in a sports club for a specific season, for example, winter or summer, and having trained in a sports club for at least 1 year.

### 2.2 Recruitment of Participants

The recruitment of athletes was performed through the Swedish Parasport Federation. An advertisement was posted on the Facebook pages of the Swedish Parasport Federation and the SO Sweden. Recruitment also took place by sending out emails to athletes who had participated in national SO competitions in recent years.

The participants who responded to the advertisement were contacted by text message or email. Thereafter, an online meeting was set up (approx. 15–30 min) where the participants were further informed and could ask questions about the study. The participants were informed about the possibility of bringing a support person to the interview, which five of the participants chose to do. The participants then signed the written consent, and a time for the interview was scheduled.

### 2.3 Participants

Fifteen athletes consented to participate in the study (female/male 8/7, 29.5 ± 9 years). They were training in a variety of clubs which comprised integrated settings (*n* = 10), included settings (*n* = 3), and both (*n* = 2). Definitions of the different settings are presented in the background under the heading theoretical framework. They were training 2–7 sessions/week, had been active in sports for 17.8 ± 8.8 years, and were active in a broad range of sports, both individual and team sports: alpine skiing, athletics, basketball, cross‐country skiing, floorball, football, handball, judo, and swimming. The level of the athletes ranged from recreational to elite. Seven athletes had participated in international elite championships such as VIRTUS World Championships or Paralympics and six had participated in the Special Olympics World Games (some had participated in both VIRTUS and SOWG). Some athletes had additional disabilities: attention deficit hyperactivity disorder (*n* = 5), autism (*n* = 3), physical impairment (*n* = 2), and Down's syndrome (*n* = 1).

### 2.4 Data Collection

Semi‐structured interviews were conducted online by two researchers (JO and MLO) during spring 2024. The interviews were adapted for people with intellectual disability with simplified direct language and consisted of five areas: (1) Who are you and what is your sport, (2) me as an athlete (goals, wishes, dreams, motivation, need for support, stress and anxiety), (3) the training (motivation for training, feelings about training, views on their trainers, thoughts on the training design), (4) safety and participation (feeling of safety physically and socially, being a part of decision making, assisting with different things in the club during training and other events), and (5) need for support (what I can manage by myself and what I need support with). During the interviews, five different colours were used in paper format—each representing a thematic area of questions—to increase clarity about the number of areas being covered and how many remained. One to four short breaks were included as needed, initiated by either the interviewer or the athlete. An ‘emotional image support’ tool was used: a sheet displaying various images representing emotions to help participants express feelings by pointing to images during emotional discussions.

The interviews were recorded using a voice recorder (Olympus, DM‐720) and at the same time automatically transcribed using Microsoft Teams version 24215.1007.3082.1590. The automatically generated transcript was thereafter compared to the voice recording and rewritten.

### 2.5 Data Analysis

The transcripts of the interviews were read through by all the authors and inductively analysed, which revealed basic psychological needs related to a sense of belonging and environmental support (in line with the PAD model). The interviews were then analysed using qualitative content analysis, focusing on the latent content using the theoretical framework of sense of belonging (Allen et al. 2021) and the PAD model (van der Ploeg et al. 2004) as a background. Two of the authors (JO and MLO) separately read through the interviews several times, and meaning units were identified and subsequently coded and sorted into categories and sub‐categories. The two authors then compared the results and discussed and rearranged the categories/sub‐categories several times until consensus was reached (Erlingsson and Brysiewicz 2017).

### 2.6 Trustworthiness

This study used qualitative semi‐structured interviews, analysed using content analysis in accordance with Graneheim and Lundman (2004). To ensure the trustworthiness of the findings, the criteria of credibility, dependability, and transferability were applied.

### 2.7 Reflexibility

Reflexibility involves the researcher's awareness of their own influence on the research process and outcomes. JO works at the Swedish Parasport Federation in sports development for individuals with intellectual disabilities. She also coaches an integrated training group (non‐paid) for athletes with intellectual disabilities in a sports club. EFW is a registered nurse and works as an applied researcher within a municipal disability support unit. KW is a researcher in special education. MLO is a researcher in Sport Science with a focus on people with disabilities, and she coaches (non‐paid) a youth team that includes athletes with and without intellectual disabilities.

### 2.8 Ethical Statement

This research was pre‐approved by the Swedish Review Authority (Dnr 2023‐08126‐01) and conducted in line with the Swedish Research Council’s (2024) guidelines for good research practice, emphasising respect, informed consent, and protection of vulnerable groups.

Special attention was given to informed consent for participants with intellectual disabilities, based on the principle that capacity should not be judged solely by diagnosis. Researchers used accessible formats and clear communication to support meaningful participation, guided by Beail and Williams (2014) and McDonald and Kidney (2012). All participants were informed of their rights, including the right to withdraw at any time without consequence.

## 3 Results

The analysis resulted in an overall theme, ‘Empower me to grow as an athlete—adapt the support to help me develop my sport skills and social aspects’. This theme was based on four categories: *I am **motivated**, I feel **competent** when I perform my sport, I **perceive** that I belong in sports*, and ***Opportunities*** *are created through support*, together with sub‐categories (Table 1) derived from the interviews.

**TABLE 1:** Categories and sub‐categories included under the main theme *Empower me to grow as an athlete—adapt the support to help me develop my sport skills and social aspects*.

Categories	Sub‐categories	Examples
I am **motivated**	Enjoyment and well‐being	My training makes me feel good both socially and physically.
	I have goals	I want others to see me as an athlete
I feel **competent** when I perform my sport	I appreciate the social aspects of sports	One of the most important aspects is the feeling of community, a sense of shared joy and familiarity with others.
	Performance and feeling of success	I like to win, but winning is not everything. I also want to feel that I have done my very best and that I have developed.
I **perceive** that I belong in sports	Like‐minded people & feeling of inclusion	I am there to be with others who share the same passion. No one is judged for their disability—there is no ‘us’ or ‘them’.
	Role model	I am or I want to be a role model to others, by being seen as an important athlete regarding both sport skills and social aspects.
**Opportunities** are created through support	Make it understandable	I appreciate when: − Social relations and rules are understandable − I understand how I can perform the activity and feel competent − Others understand me.
	Develop my sport skills	I want to be challenged in the activity. Do not set limits on my development. Listen to my goals and support me to understand how to reach them.
	Improved athlete pathways	There are not enough opportunities for me as an athlete.

The results are presented below with quotes illustrating the categories and sub‐categories. To protect anonymity, the specific sport has been anonymised, and the term international championship is used instead of VIRTUS World Championships, the Special Olympic World Games, or Paralympic Games.

### 3.1 I Am **Motivated**

The category *I am **motivated*** emerged as an important area for the respondents with sub‐categories *Enjoyment and well‐being*, and *I have goals* (Table 1). This category explored the athletes' motivation for participating in the sports activity and reflects the importance of enjoyment, their wish to develop their sport skills, that they have goals related to both performance and well‐being, and appreciate when coaches ask about their goals.

The sub‐category *Enjoyment and well‐being* reflected the importance of enjoyment, that the activity is fun, which was mentioned by almost all of the athletes.

… but it's so much fun, and that's mostly why. Also, you want to grow and develop as well. (Participant 3, female)

The sub‐category *I have goals* represented the importance of having someone who cared about their goals and dreams, and that they were listened to and respected. When asked about their sporting careers in 5 years, 10 athletes responded quickly with clear performance goals:

I have maybe hopefully won an international championship medal and some more merits in my sport and that there are more international competitions. (Participant 14, female)

### 3.2 I Feel **Competent** When I Perform My Sport

The category *I feel **competent** when I perform my sport* emerged as an important area for the respondents, with sub‐categories *‘Appreciate the social aspects of sports’* and *‘Performance and feeling of success’* (Table 1).

The sub‐category *‘Appreciate the social aspects of sports*’ emerged from the fact that almost all of the athletes highlighted the sense of community as one of the most positive aspects of their sports club. They emphasised the feeling of belonging, the opportunity to share joy with others, and to be in an environment where no one is judged based on their disability.

My club is about community, where everyone gets to be involved, and what I'm passionate about is making sure that everyone is seen and receives help, without judging anyone because of their disability. (Participant 8, female)

The sub‐category of *Performance and feeling of success* was informed by the feeling of wanting to win, enjoy winning and being the best as an important goal.

My goal is to win a gold medal at an international championship, where I compete in my sport. (Participant 3, female)

Because I want to win and claim my position. (Participant 13, female)

The athletes also reflected on the importance of winning games, races, or competitions, while acknowledging that it can equally be about embracing who you are and performing in accordance with your own abilities, without comparing yourself to others.

Oh, I think it's good that you can be who you are, right? I mean, work according to your own ability, right? And just be yourself when you step out. Instead of, like, comparing yourself to someone else. (Participant 5, male)

Winning a medal does not always guarantee a feeling of success; rather, the most important factor is to feel that you have given your best performance. Success for the athletes is about gaining a sense of having performed at their best, for example, achieving a personal best, even without winning a medal.

But the real win for me is that if I do my sport faster than I've ever done before, if I set a personal best and stuff like that, that's the real victory for me. The medals matter less. (Participant 14, female)

### 3.3 I **Perceive** That I Belong in Sports

The category *I perceive that I belong in sports* was formed of two sub‐categories: *Like‐minded people and feeling of inclusion* and *Role model* (Table 1). When being together with others in similar circumstances (like‐minded people) allows the athletes to feel a relation to what others are talking about, which gives a feeling of a sense of belonging.

Everyone is friends with each other—we spend time together during training and have this sense of familiarity with what most others are saying. (Participant 4, female)

The feeling of belonging in a sports club can create a sense of community beyond one's own training group or team to include other training groups and teams within the club or other clubs. The feeling of inclusion relates to a sense that there is ‘no us or them’.

What I think is good about our club is that everyone is so included in the club. There's no ‘us or them,’ the youth teams greet us para‐athletes just as much as anyone else. (Participant 15, male)

As many sports are transferring from a disability‐oriented club (segregated setting) to a sport‐specific club (included or integrated setting), athletes perceive that this transition have increased the feeling of inclusion also at competitions. Before in the segregated setting, the feeling was that athletes with disability were cheering on other athletes with disability and not anyone else. With the club's transition to an included/integrated setting, this had changed.

I mean that we can compete, and that everyone is cheering each other on, you know? (Participant 7, male)

The sub‐category *Role model* relates to the feeling of being or wanting to become a role model for others. Being a role model for others seems to be a benchmark for success. The perceived attributes of a role model were multi‐faceted, such as performing, winning, and receiving congratulations from sports club friends. Another role model attribute was the ability to inspire others through hard work and fair play. Athletes aspired to be able, in the future, to look back at their own careers and know that they had inspired others in Para sports. A role model was also identified as having the ability to inspire others to show emotions and be honest about how training can impact well‐being both physically and mentally.

To kind of inspire others about how it feels—how it feels when you finish a training or how—how you feel afterwards if you don't. Yeah, because then life feels more meaningful for me [when active in sports], to be the person I am today. (Participant 2, male)

### 3.4 **Opportunities** Are Created Through Support

The last category, *Opportunities are created through support*, was an area where the athletes highlighted the coach's role in providing support, with the sub‐categories *Make it understandable*, *Develop my sport skills*, and *Improved athlete pathways*.

The sub‐category *Make it understandable* highlights the importance of understanding the communication between athlete and coach and also between athletes. It was shown that understanding was important for enjoying the activity, and that adapted support from coaches or others was crucial in order to achieve this. Understanding involved not only direct instructions on activities during training, equipment, and schedules, but also support regarding social and emotional aspects.

Using assistive tools for communication and instruction made training easier. Several athletes mentioned using mobile phone applications (non‐disability specific) to check training times, locations, and training plans by themselves.

No but we have this kind of app, where it says that training is from 19:30‐21:00. (Participant 2, male)

Schemes with image support for the week and the specific day are often used by people with intellectual disability both privately and at work to make things easier to understand and remember. Clear information helped the athletes understand aspects of the training beforehand and also other things such as what to do if they were unable to attend the training.

We do get a monthly schedule. With the time, place and the coach in charge, and their phone number. If I can't go, I need to text them myself to let them know. (Participant 9, female)

Many athletes valued that the coach's instructions were short, with clear language and visual representations of body movements. Pedagogical support such as film clips, writing on a board, and image support during trainings were appreciated. However, schemes for the month, week, and training session were only used by a few athletes.

The athletes also mentioned that sometimes they do not understand what the coach is instructing them to do and what they are supposed to do during the training. When this happens, they develop a strategy of mimicking others.

If I don't understand the training we're supposed to do in that situation, then I let someone else go first who understands, then I can see how it's done and follow them. (Participant 5, male)

Communication relies on mutual understanding, and athletes with intellectual disability may struggle to express when something feels too difficult. It may also be particularly difficult to ask the coach for clarification, in terms of formulating the request. This can be a significant challenge and may lead to frustration.

I thought it was too difficult. I just got frustrated.

Interviewer:

Did you say anything to the coaches when you couldn't get it right?

*‘No’. (Participant 10, male)*.

Regarding understanding and development of social aspects on the group level, it was appreciated when the coach would step in, gather the team, and talk to the athletes about a bad social atmosphere. This was considered important for building a strong sense of unity.

So there have been times when the atmosphere hasn't been the best, you know, but it's been much better now since the coaches talked to us about how to act, and stuff like that, so it's much better now, yeah. (Participant 3, female)

Support with social aspects on the individual level was also important. Some athletes could experience feelings of ‘I do not want to go to the training’, for example, if the day had been tiring already before the training. Having a supportive check‐in with the coach, covering how the athlete is feeling and the plan for the training, was highly appreciated by the athletes. This approach can reduce resistance to attending training and help calm down the athlete by providing clarity about what to expect.

But the coach and I keep in touch. Sometimes we check in, like, how are you feeling ahead of tonight's training? This is the idea, will that work for you, and so on. (Participant 6, male)

Social support is also needed when attending competitions or camps, as athletes with intellectual disability may feel stressed by busy schedules with different activities and have unclear expectations, even with prior verbal information. Lack of preparation results in frustration and reduces the enjoyment.

… and I really wanted to be able to enjoy being at the competition without feeling so much stress. Especially in this big competition, we had a tough schedule. We had to get up early—like, it was insane, according to my mum. We even had to get up at 5:45, and then breakfast was at 6:30. … It was one of the most stressful moments of my life. (Participant 2, male)

The sub‐category *Develop my sport skills* tells us that the athletes want to be challenged to develop as athletes.

[I appreciate] that they tell me what to do during the trainings, what I can improve. (Participant 7, male)

The trainings are most often performed in groups. The number of athletes in the training group can be challenging in two respects: it can be hard to concentrate if the group is too large; and if there is a wide range of skill levels among the athletes, it can lead to a feeling of no development. For athletes to be able to develop and reach their personal goals, it is essential to be challenged within one's sport. The athletes mentioned frustration when repeating the same activities too much, which was perceived to stall their progress—producing a feeling of being coddled and that coaches have preconceived notions about what the athletes can achieve just because they have disabilities. Also, when requests for a higher level of challenge are ignored, it feels frustrating and disrespectful.

Then you'd have to go and talk to them again and they'd listen but then… You'd feel that tightness in your chest when you had to bring it up again. And say that we need some different drills, or it's just the same thing over and over. (Participant 8, female)

Working with goals was highlighted as a facilitator for developing sport skills and supporting a sense of belonging in sports—both being asked about their goals and receiving support on how to train to reach them.

Yeah, I have pretty good coach support. So I can [reach my goals]. I normally train and then I talk about what I need to work on and what I can improve. (Participant 2, male)

On the other hand, not being asked about their goals and wishes was a disappointment. One athlete describes a situation where she had to bring the subject up herself.

That you talk about, what personal goals do you have? We never really had that. You could mention it a bit yourself, but they (the coaches) never came and asked,

‘What are your goals, and what do you want to continue with?’ It was mostly that you yourself said,

‘This is what I want to do.’ (Participant 8, female)

Support regarding feelings related to winning and losing also emerged as important. The athletes appreciate support by the coach to develop their views on performance and psychological development so that even if they lose, they are supported to feel that they have still done their best based on the circumstances.

You win and lose, that's just something you can't do much about, right? The thing is, that's what life is about, you can't always be the best. I get that. I have really good support, they said. Someone said that I'm the best because you did your best. (Participant 2, male)

The last sub‐category, *Improved athlete pathways*, reflects that athletes with intellectual disability also dream of a sport career pathway, for example more competitions generally, and opportunities to go to training camps, compete at national or international level, or be a part of the national team. However, limited pathways, hinder their ability to reach these goals.

I want to be the best player I can be, given my circumstances. I have even dreamed of… I don't know if it's possible but to play in [an international championship in their sport]. Unfortunately, the [national federation] isn't quite there yet. (Participant 15, male).

## 4 Discussion

The aim of this study was to explore motivation for engaging in sports among athletes with intellectual disability using the theoretical framework of sense of belonging. A secondary aim was to explore athletes' need for support. The results revealed the overarching theme *Empower me to grow as an athlete—adapt the support to help me develop my sport skills and social aspects* when using the theoretical framework of sense of belonging (Allen et al. 2021) and the PAD model (van der Ploeg et al. 2004) as a background. The main theme of the current study—sport skills and social aspects—echoes perspectives on sport participation among athletes with intellectual disabilities from a study done in the USA, where training in sport and social connectedness emerged as central themes (Weiss et al. 2017).

The results of the present study showed that the sense of belonging in the sport context was related to athletes *having motivation to participate* and *feeling competent* regarding both sport skills and social aspects. The study found that athletes are *motivated* to participate in sports because the activity is enjoyable and gives a feeling of well‐being relating to both physical and social aspects. The social aspects include a shared enthusiasm for the sport with others and the opportunity to train together with like‐minded people with whom they feel coherence and a sense of familiarity, in a socially safe environment where no one is judged for their disability, reflecting a sense of belonging in sports (Gur and Bina 2023). In a newly published review article by Evans et al. (2025), it is demonstrated that social inclusion is fostered by inclusive environments that provide social interaction, joy, and opportunities for achievement. This yields similar results as in the present study, showing categories of sense of belonging from both social aspects and sport competence. The feeling of sport competence has been linked to motivation and participation in adolescents without disabilities, where self‐perceived sport competence is related to intrinsic motivation (Bagøien and Halvari 2005). Athletes in the current study expressed having goals and dreams for their sport, but these were rarely discussed with coaches or other athletes, despite a desire to do so. This may reflect a situation where coaches view them more as participants engaging in sports recreationally for enjoyment rather than as athletes striving to improve their skills. Similarly, coaches in another study put greater emphasis on social interaction, positive emotions, and life skills regarding athletes with intellectual disabilities, while focusing more on performance‐related goals for athletes without disabilities (Sakalidis et al. 2023).

The category *I perceive that I belong in sports* was also closely related to possessing an athletic identity, encompassing both sport skills and social aspects. Regarding sport skills, this was related to being competent and able to follow the training, having opportunities to develop and perform as athletes, and achieving success. These results are in line with Brewer et al.'s (1993) theory of Athletic Identity, which highlights that having an identity as an athlete fosters a positive self‐image and provides a meaningful context where individuals ‘feel competent at something’. It was mentioned as negative when the activity was too easy, which could lead to a feeling of stagnation and of being coddled, and that the coach did not believe in the athletes' development of skills. The reason for making training too easy (underloading) may be due to coaches' attempts to protect their athletes, but it instead resulted in frustration, limited development, and a sense of exclusion, as the athletes are not given the same opportunities to progress as those without intellectual disabilities (Svanelöv et al. 2020).

The social aspects of *perceiving belonging in sports* related to being together in a training group with *like‐minded people* who enjoy sport, who are friends with and care for each other. This fosters a *feeling of inclusion* in the sport community where, as cited in the results section, ‘there is no us or them’ and ‘everyone is cheering each other on’. Perceiving belonging in sports occurs when the athletes with intellectual disability are a real and natural part of the sport community like everyone else, and are listened to and respected. Similar results were found in another study where sport participation enabled people with disabilities to develop an identity as athletes, rather than being seen simply as people with disabilities (Pack et al. 2017). Being or wanting to be a *role model* to others was perceived as a strong indicator of the feeling of being an athlete and belonging in sports. It was important to the athletes that others saw them as having both good sports skills which contribute to performance/success and an important social role as someone who supports others and contributes to the social climate.

The results of this study showed that athletes with intellectual disability feel a sense of belonging in sports clubs, and *opportunities* for participation are created when adapted support is present. Adapted environmental support is tailored for individual needs according to disability‐specific personal factors (Burns and Johnston 2020; Cunningham and Warner 2019). The athletes relate the environmental support mainly to the actions performed by the coach, affecting both themselves and the group of athletes they train with. The category *Opportunities are created through sport* was created from the sub‐categories *Make it understandable, Develop my sport skills*, and *Improved athlete pathways*. When the coaches make the activity understandable, regarding both sport and social aspects, it creates a secure and safe environment that supports feelings of competence and development of sport and social skills. The athletes also appreciate when the coach takes steps to make the social climate understandable and supports the climate to develop. This happens, for example, when the coach talks to the whole team about how the team can support each other and gives examples of how to act to develop the social climate. Establishing meaningful relationships with athletes is a crucial component of coaching that enables the development of trust‐based connections, which in turn leads to a safe, supportive environment (Aspelin 2010). Applying a relational pedagogy—based on human interaction and the importance of relationships in learning—is central to this approach (Aspelin 2010). The importance of the coach‐athlete relationship emerged in the current study, as the athletes particularly valued when coaches engaged in relational practices such as sending encouraging text messages, checking in on their well‐being, motivating them to attend the training, and explaining what the training would include in advance.

A part of intellectual disability is having difficulties with abstract thinking and memory capacity, reducing the ability to pre‐conceive and visualise what will happen and remember information (Levén et al. 2011). In the current study, these personal disability‐specific factors also emerged as difficult aspects in the sport context, highlighting the lack of environmental support according to the PAD model (van der Ploeg et al. 2004). The athletes described using a weekly/daily schedule with image support in private life to help them recall and comprehend daily activities; however, such tools seemed to be seldom used in the sport context. Therefore, implementing more visual support strategies within the sports context could reduce the barrier of limitations in abstract thinking and memory capacity and thereby facilitate understanding and enhance participation for athletes with intellectual disabilities.

All the interviewed athletes, whether more recreational or elite athletes, had a wish to develop in their sports. They generally raised that they appreciated when and how their coach supported them in this process, regardless of whether the goal was a recreational goal related to well‐being or a performance‐related goal to achieve success. Within the dream of being an athlete, the results also revealed the wish for an improved sport pathway for those with intellectual disability. The athletes dreamt of more opportunities to attend competitions and training camps. Many of the athletes were aware that athletes without disabilities in their sport had different pathways and, for example, could aim for the national team, while for them as athletes with intellectual disability, there is no national team or national championship in their sport. Some athletes were aware and grateful that their coaches were supporting the development of the sport context also on the organisational level, adding classes for athletes with intellectual disability into existing competitions and camps.

The current study showed the feasibility of inclusive research practice using online interviews for persons with intellectual disability using adaptations. The efforts of adaptations of image‐supported information, having a preliminary meeting prior to the interview, the possibility to bring a support person, and breaks during the interview were made to enhance understanding, focus, social safety, and reduce stress for the participants. Several participants said after the interview that they sincerely appreciated being a part of this study, being asked about their feelings, and being listened to.

## 5 Limitations

A challenge in this qualitative study relates to the communication and the interpretation between the researchers and the athletes. The adaptations were included to increase clarity, with the awareness that adaptations can disturb the participants' own voices. For example, the researchers focused on letting the participants speak on their own, and only they could ask the support person to fill in and explain. The interviewers were not familiar with sign language, and for one athlete, the support person translated the sign‐supported talking of the athlete. The interviewers both had pre‐understanding of training athletes with intellectual disabilities but not from the same sport, club, or athletes. This increased the researchers' understanding of when to ask follow‐up questions and see the need for breaks.

Another limitation of this study concerns the recruitment method through channels of the Swedish parasport federation. Participants themselves or coaches and support persons answered the advertisement of the study from either social media channels or e‐mails. This implies that the participation was self‐selected and may therefore have resulted in a relatively narrow and potentially biased sample, both regarding the level of intellectual disability and ability for written and verbal communication. For future studies, it might be worth considering sending invitations to all sports clubs in Sweden to obtain a broader sample of participants.

## 6 Conclusion

The study is rare and important because it centres on the voices of athletes with intellectual disabilities themselves. The overarching theme ‘*Empower me to grow as an athlete—adapt the support to help me develop my sport skills and social aspects*’ emerged from athletes with intellectual disability feeling a sense of belonging in the sports context. The athletes with intellectual disability are motivated, want to feel competent in their sports, and to feel like athletes who belong in sports and develop as such. They also appreciate the adapted environmental support given by coaches regarding both sport and social aspects, promoting a more inclusive sport community. The athletes expressed a need for the coaches to enable opportunities for a sense of belonging by adapting their support to make the sport context understandable, focusing on helping athletes develop their sport skills, and trying to improve athlete pathways. The results of this study can assist coaches and federations in developing their sport to enable athletes with intellectual disability to increase their feeling of belonging in sports.

## Author Contributions

All authors together conceptualised the study. J.O. and M.O. conducted the interviews and analysis. K.W. and E.F.W. acted as critical friends in the analysis. All authors reviewed the results and contributed to their interpretation. J.O. wrote the initial draft of the manuscript. All authors contributed to the writing of the manuscript. All authors reviewed and approved the final draft of the manuscript.

## Disclosure

The authors have nothing to report.

## Ethics Statement

This research was pre‐approved by the Swedish Ethical Review Authority (Dnr 2023–08126‐01) and conducted in line with the Swedish Research Council's (2024) guidelines for good research practice, emphasising respect, informed consent, and protection of vulnerable groups.

## Consent

The authors have nothing to report.

## Conflicts of Interest

The authors declare no conflicts of interest.

## Data Availability

The data that support the findings of this study are available on request from the corresponding author. The data are not publicly available due to privacy or ethical restrictions.
